# Brain aging: more of the same!?

**DOI:** 10.18632/aging.101799

**Published:** 2019-01-29

**Authors:** Elisabeth J. Vinke, M. Arfan Ikram, Meike W. Vernooij

**Affiliations:** 1 Department of Epidemiology, Erasmus MC, Rotterdam, the Netherlands; 2 Department of Radiology and Nuclear Medicine, Erasmus MC, Rotterdam, the Netherlands

**Keywords:** brain aging, neurodegeneration, longitudinal, trajectories

With the number of patients suffering from brain diseases set to soar in coming decades [1] it has become of great importance to not only understand normal functioning of the brain but also unravel when, how, and where in the brain deviations take place from the path of normal aging towards pathological degeneration and ultimately clinical disease. A first essential step is to map normal aging trajectories of the structure and function of the brain. This research question is as simple in its statement as it is complex in its operationalization.

Magnetic resonance (MR) imaging has been the single most important contributor to the in vivo investigation of brain structure and function. Over the last three decades, an ever-increasing number of imaging datasets have become available capturing every phase of the human lifespan. With these datasets, trajectories of brain imaging markers in an aging population can be estimated, based on a single brain scan per subject. With a single measurement, taken at a single moment in time, merely a snapshot of the dynamic process of aging is taken. Considering the high inter-subject variation, investigating what brain aging is with only cross-sectional data is challenging.

While these cross-sectional studies have laid an important and solid foundation, to investigate brain aging in more depth and to better discern between patterns of normal versus abnormal aging, longitudinal data is essential to provide better insight into the timing and sequence of changes in aging [2]. Compared to cross-sectional studies, with longitudinal data one is able to investigate when and how the deviations from normal aging occur, rather than the average absolute differences between young and older subjects. Distinguishing different trajectories based on longitudinal data can be a starting point to study why certain persons show a different aging pattern than others, which factors drive these differences, and what functional outcomes these relate to.

A frequently used approach to investigate different patterns of changing imaging markers over time, is to simply subtract measurements from two different time points to identify subjects that increase, decrease or remain stable over time. Once the subjects belonging to each of these categories are identified, comparing the population characteristics between these groups could point towards potential factors influencing the trajectories. Although this approach could be an important first exploration, we believe that this use of longitudinal data is not living up to its full potential. First, classification of subjects into these three categories based on only two longitudinal measurements could be very sensitive to noise and therefore lead to misclassification. Second, the simplification of the course of the trajectories makes it less sensitive to detect more subtle slope differences. Third, though in this approach the slope of the subject-specific trajectories is taken into account, the age on which these slope differences occur is not.

**Figure 1:**
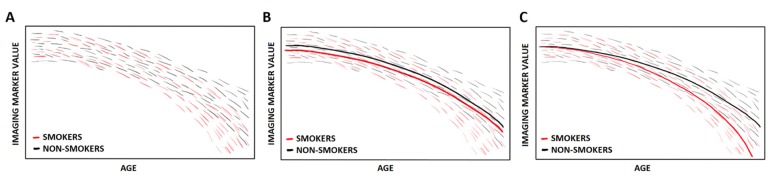
Fictional subject-specific trajectories of an imaging marker in aging, from smokers and non-smokers (**A**), with population-based trajectories assuming that every subject follows the same trajectory only allowing for a different starting point (**B**) and population-based trajectories with smoking as an effect modifier (**C**).

To give an example of how one can use longitudinal data to further explore different patterns of imaging markers, assume the hypothetical situation that we have identified using the method described above a potentially important factor that could influence the aging trajectory: smoking. Figure 1A shows fictional subject-specific trajectories of smokers and non-smokers for a specific imaging marker. We can then use the subject-specific trajectories to study whether and how the differences in the trajectories are explained by smoking. Instead of assuming that everyone follows the same aging trajectory, only allowing for a different starting point (Figure 1B), approaching smoking as an effect modifier of the effect of age on the marker is a way to capture more subtle changes in the shape of the trajectory (Figure 1C). Even though everyone follows their own trajectory, the effect of smoking can be determined by estimating to which extent each subject-specific trajectory is explained by smoking. Combining all that information gives an overall approximation of the effect of smoking, that would have been missed or underestimated when simply assuming that everyone has the same shape and that smoking only influences the intercept.

Next to the ability to investigate different trajectories of a single imaging marker, longitudinal data on not just one, but several markers within the same subject are essential to assess how changes in these markers coincide and interact, and to assess the temporality of these events. In our recent work we investigated the trajectories and sequence of changing structural brain imaging markers in a large aging population, using longitudinal brain imaging data [3]. The resulting sequence of changing markers could be interpreted as an average sequence of the broad spectrum of normal aging in this population. We believe that within aging research, the field can take example of research performed in the setting of diseases with a very heterogeneous clinical presentation, such as multiple sclerosis. In these diseases, timing of events such as the onset of certain symptoms or presence of disease markers are already being used to investigate and identify subtypes of disease and to predict progression of disease. Considering that we may never be able to draw a clear line between normal aging and abnormal aging, we believe that with a special focus on the timing and sequence of events in brain aging, we may also be able to identify different patterns of aging in a similar way. This could greatly advance research into brain health in old age.

To summarize, more imaging data on the same subjects gives us the opportunity to focus on timing and sequences of changes, which can help us to identify different patterns within the broad spectrum of normal aging. This would bring us one step closer towards understanding the sources of variability, and their implications, within ‘normal’ aging.
